# A Review on Potential Issues and Challenges in MR Imaging

**DOI:** 10.1155/2013/783715

**Published:** 2013-11-27

**Authors:** Srinivasan Kathiravan, Jagannathan Kanakaraj

**Affiliations:** PSG College of Technology, Peelamedu, Coimbatore 641 004, India

## Abstract

Magnetic resonance imaging is a noninvasive technique that has been developed for its excellent depiction of soft tissue contrasts. Instruments capable of ultra-high field strengths, ≥7 Tesla, were recently engineered and have resulted in higher signal-to-noise and higher resolution images. This paper presents various subsystems of the MR imaging systems like the magnet subsystem, gradient subsystem, and also various issues which arise due to the magnet. Further, it also portrays finer details about the RF coils and transceiver and also various limitations of the RF coils and transceiver. Moreover, the concept behind the data processing system and the challenges related to it were also depicted. Finally, the various artifacts associated with the MR imaging were clearly pointed out. It also presents a brief overview about all the challenges related to MR imaging systems.

## 1. Introduction

At one time, assembling a spectrometer was the first step in beginning NMR research. Bloch et al. [1] and Purcell et al. [2] soon after the description of the chemical shift phenomenon created a market; however, companies such as Varian and Oxford Instruments began to manufacture spectrometers and magnets. The result was that researchers were able to channel their energy into pulse sequence development and applications rather than hardware design. MRI began similarly with early whole body systems such as Damadian's “indomitable” built from scratch in a laboratory until first EMI, and then other companies began producing integrated imaging systems. By the late 1980s MRI research was performed predominantly with commercial systems designed for clinical scanning.

The limitations and inflexibility of these clinical systems lead to the development of after-market spectrometers and hardware upgrades from companies such as SMIS and ANMR. The 4 T human magnet has never been in widespread use. The milestones are 1.5 T, 3 T, and 7 T. As it stands in the early 21st century, aside from corporate development labs and a few isolated university sites, MRI hardware has become more of a commodity than a subject of research. This was, perhaps, inevitable, as NMR has become a tool for physicians and scientists rather than just a discipline in and of itself.

Despite the current reduced emphasis on facility with spectrometer hardware among researchers in MRI, successful implementation of high field systems demands an investment of engineering resources. Most of the subsystems in an MRI scanner: magnet, gradient, radiofrequency front end, spectrometer, radiofrequency coils, and patient handling require some degree of redesign and adaptation to function optimally at high field. While the art of engineering is most apparent in the realm of radiofrequency coils, each subsystem has its own issues, stemming directly from the increased field or frequency.

## 2. Magnet Subsystem

The limiting factor in high field MRI has always been magnet technology. The challenge for designers has been to increase the field strength achievable with a large bore magnet without sacrificing spatial homogeneity, temporal stability, or patient access and without exorbitantly increasing magnet weight, cost, and fringe fields.

**Figure 1:**
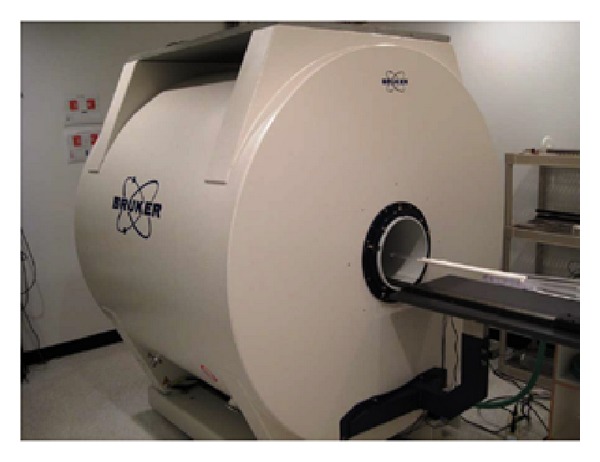
7 T Bruker Avance magnet.

Figure 1 illustrates 7 T Bruker Avance magnet. The magnet is enclosed in an RF shielded room to minimize noise from outside RF electromagnetic fields. Radio frequency (RF) coils are used for signal excitation and reception.

### 2.1. Magnet Components

An MRI scanner is based on a large electromagnet, which produces the main static magnetic field described in the previous section. The magnet is the main factor in determining the cost, appearance, and capabilities of the MRI system. The main characteristic of the magnet is the strength of the magnetic field produced within the patient. Also important are the uniformity and temporal stability of the magnetic field. A magnetic field is generated either by using a ferromagnetic material or by passing an electrical current through a wire. The former method is more efficient and produces less stray fields, but it has the disadvantage of having limited design flexibility and is very heavy. Most MRI scanners use the electrical current as a source of magnetic field. The simplest design is the solenoid, which produces large volumes of uniform field and is very flexible. The resistive or superconductors are the two types of conductors used. The resistive magnet is simple and has a low cost to construct; however it only produces small regions of low field strength when considerable power is used. Superconducting magnets are more difficult and expensive to construct, but they produce large volumes and high fields, Felix et al. [3].

A modern superconducting magnet system is made up of four parts: the main superconducting windings, the cryostat, the superconductive shims, the quench circuitry, and the magnetic shielding. Strong static magnetic fields for NMR and MRI can be created with three different technologies: resistive electromagnets, permanent magnets, and superconducting electromagnets. Permanent magnets, which do not require an external power source, are made from “hard” magnetic materials, which are difficult to magnetize but once magnetized maintain their magnetization for a long period. While permanent magnets are coming back into favour for low field open systems, they cannot practically be built for high field applications. Resistive electromagnets predominated in early NMR and MRI systems. They require current flow in metallic wires to generate a magnetic field without the use of any magnetic materials. They are inexpensive and are simple to construct but generate heat due to the currents in the windings.

However, the power required to run those increases substantially with field strength. Resistive magnets for whole body imaging were therefore impractical at fields above about 0.18 Tesla, corresponding to a continuous power consumption of 40 Kilowatts, Black et al. [4]. Superconducting electromagnets have now replaced resistive electromagnets as the technology of choice for MRI. Superconducting magnets require no external power source once energised and have no resistance when cooled to below their transition temperature. These magnets are currently widely used in MR scanners producing fields between 0.15 and 4 Teslas. The magnets up to 7 Tesla are commercially available.

These magnets hold a magnetic field by trapping a transiently applied current in a continuous loop of superconducting wire. Hence, once energized such magnets are persistent and only require refilling of their cryogens to maintain their superconductivity and their field. The advantages of superconducting as compared to resistive magnets are substantial: in addition to making fields above 0.2 Tesla possible, the field generated by a superconducting system can be larger in extent, more uniform, and more stable without significant ongoing power consumption.

All of the above magnets and hybrid magnets have been successfully used for MRl. The superconducting magnets have dominated MRI since the mid-1980s, due to their ability to reach high field strengths and achieve a high degree of field homogeneity. Superconducting magnets need cooling to reach their critical or transition temperatures. This is achieved by using liquid Helium as the refrigerant, where considerable effort is made to minimize heat loss from the Helium vessel by reducing the number of conduction, convection and radiation paths, Webb [5]. A typical superconducting magnet has about 17000 turns of wire with a radius of about 0.65 m. The wire and its supporting structures weigh about 3 tons.

Superconducting wire is made from either multiple filaments, a solid core of a type II superconductor such as niobium-titanium alloy inside a copper matrix, or wire in channel technology. Despite higher cost, high field systems were generally made with filamentous wire due to lower joint resistance and better heat dissipation. Wire in channel is now being used for ultra-high field large bore systems because its strength resists the loop forces, which can exceed 21,000 pounds per square inch in a modern magnet, better than filamentous constructions. Niobium-titanium alloy becomes superconducting only at temperatures below 9.8 K, and so, regardless of the wire type, the windings must be cooled with liquid helium. A cryostat filled with liquid helium at 4.2 K contains the windings and an outer vacuum bottle minimizes conduction of heat from the magnet's surroundings. Within the outer portion of the vacuum bottle, surrounding the cryostat, there are either a set of cooled radiation shields or an outer cryostat filled with liquid nitrogen at 70 degrees Kelvin depending on the particular design.

Cryogenic cooling of superconducting magnet windings has also changed in technology. Due to the high cost of liquid helium and demand in availability, it should be handled with utmost care. Helium undergoes a phase change at relatively low temperature and gets transformed into a gas which then gets dissipated. Efforts must be made to transfer any heat absorbed by the helium into some other material to limit helium boil off, since it is needed to cool MRI magnets that work at high temperatures due to its superconductivity and high energy current. Early magnets contained the liquid helium cryostat inside a liquid nitrogen cryostat. Thus relatively cheap liquid nitrogen was boiled off rather than helium. More modern magnets do not contain liquid nitrogen. Instead, they remove heat from the helium via a refrigeration system. These “cold head” refrigeration systems typically use helium as their refrigerant and operate on the principle that compressed liquid helium cools as it expands and evaporates, thereby creating a gradient through which it can absorb heat. These cold heads are mounted to the radiation shields surrounding the cryostat so that they remove any accumulated heat. An alternative design is a full helium refrigerator, which collects gaseous helium which has been boiled off from the cryostat, compresses it, and recycles the liquid back to the cryostat.

- Magnetic shielding: since lines of magnetic flux must close upon themselves, it follows that to create a strong magnetic field inside the magnet bore, a significant magnetic field must also exist outside the bore. While field strength falls off with the square of distance, these fringe fields remain quite strong in the case of high field systems. In order for a 4 Tesla field to fall off to 5 Gauss, the level at which cathode ray tubes and pacemakers are no longer interfered with, the field must be reduced by a factor of 8,000 by shielding systems. This may be accomplished with active or passive shielding methods. In active shielding, another set of superconducting windings with opposite polarity are arranged in series with but physically outside of the primary windings.
- This also has the effect of reducing the field inside the bore to some degree, and thus active shielding is not typically used in very high field designs. The second shielding method, passive shielding, uses iron, or another highly permeable material such as mu metal to capture the magnetic field within a well defined region. The passive shielding material may be affixed to the magnet itself or, more commonly, is constructed into the walls of the room enclosing the magnet. Passive shielding directly on the magnet obviates the shielded room but greatly increases the weight of the magnet and complicates installation. Any form of passive shielding which is not symmetric, however, will distort the main field and will require shimming to restore homogeneity. This can occur when the magnet room has a door only on one side or when holes must be cut in the ceiling to elevate cryocooler heads into a region of lower field. Neither technology completely contains the fringe fields in very high field installations, however, and so adequate space must be devoted to the magnet site to prevent interference with neighbours.
- Field shimming: homogeneity of the magnetic field is achieved with at least four layers of correction of the field produced by the main windings. The first layer of correction is designed into the magnet itself in the form of higher order windings. Typical superconducting magnets use a Helmholtz pair of superconducting coils to create a roughly homogeneous field over about a 40 or 50 cm diameter spherical volume. The pincushion effect such a pair creates can be corrected with another pair of windings axially and radially outside the first. Higher orders can be used as needed. In the limit, a full length solenoid creates the most homogeneous field. Notwithstanding the material cost in wire and large number of superconducting joints that must be made in order to form such a long winding, the latest ultra-high field magnets use solenoid construction.
- Whether built from discrete coils or one solenoid, the main winding sets the base homogeneity according to its design. In order to smooth the field, magnets have discrete superconducting shim coils within the helium dewar, the current in each of which can be individually adjusted when the magnet is first energized to maximize field homogeneity. The different superconductive shim coils have a variety of geometries that allow them to counteract variously shaped inhomogeneities in the main field. Typically based on Golay or Maxwell pairs, a full set of shims includes up to fourth order correction fields. Due to the direct coupling with the main windings, Z^2^ and Z^4^ superconducting shims experience damagingly high forces and currents during an abrupt quench and are thus no longer included in large bore ultra high field magnets.

After the superconductive shims, the next layer of shimming is another semipermanent system: the passive shims. Passive shims correct high order inhomogeneities by distorting the main field with ferromagnetic washers. Passive shims consist of a set of shim trays arranged circumferentially around the bore as part of the gradient insert or a separately inserted shim set. These washers can be mounted at defined intervals along each shim stick such that they counteract inhomogeneities that are too irregular to be smoothed by coils.

The final layer of the shim system is the resistive or room temperature shims. These are electromagnetic coils, similar in geometry to the superconducting shims, energized by a direct current power system. The room temperature shims, unlike each of the other layers, can be adjusted to optimize homogeneity for a particular subject or even a region of a particular subject. Using any number of algorithms, the resistive shims can be used to shim out distortions in the field caused by the structure of a particular sample.

### 2.2. Issues in Magnet

There are several barriers to high field magnet construction. The forces on a superconducting solenoid and the formers it is wound on are substantial. Not only must the magnet structure resist deformation but also even small movements of the wires must be prevented. When the Lorentz forces accumulated as a magnet is ramped up cause wire movement in the windings, that movement in the presence of friction will generate heat that can potentially raise the wire above its critical temperature and cause a training quench in the magnet as a whole. To prevent this, the windings must be laid in narrow channels of high heat conductive material. The use of conductive channels has the added advantage of safely dissipating heat in a quench so the magnet is not damaged.

The ultimate barrier to high field magnet construction is not training quenches but rather the limit on flux density that the wire can support before losing its superconductivity. For the current wire of choice, niobium titanium, the critical field is about 9.4 T at 4.2 K. This critical field may be lowered to about 12 T by pumping on the helium to lower its temperature to 2 K. Beyond that limit, succeeding generations of magnet will have to use a different superconductor such as niobium-tin alloy.

## 3. Gradient Subsystem

### 3.1. Purpose

Within the magnet, there are three gradient coils, each with a separate power supply and independent computer control, which produce the gradient fields allowing slice selection and encoding of spatially dependent information into the MR signal. Many coil shapes can be used, but in MR scanners using superconducting magnets, it is common to wind all three coils into a cylinder surrounding the patient. The main properties of the gradient coils are to make the magnetic field a function of position. The effects of the gradient fields should be strong enough to overcome the effects due to inhomogeneities of the main applied field. However, high gradient fields are difficult to produce and switch quickly and need a bigger receiver bandwidth for data collection, which in turn lowers the signal to noise ratio of the received signal. Gradient coils must also produce fields with high linearity, which controls the accuracy of the collected data. Nonlinearity of the gradient fields results in spatial distortions of the image. Gradient fields must have a short rise time (about 1 msec), which is achieved through a complex process since the gradient coils are placed in the middle of a magnet. Due to the switching of gradient fields, Eddy currents which oppose the fields produced by the gradient coils are produced in surrounding materials, resulting in poor image quality. To reduce the effect of the Eddy currents, the gradient coils are driven by complex waveforms that depend on the type of magnet and imaging sequence being used.

The gradient subsystem is designed to create linear inhomogeneities in the static field for the primary purpose of spatial encoding. In the general case, it consists of a set of three orthogonal electromagnetic coils energized by high power constant current amplifiers. These three coils each create a gradient in the static field that is directly proportional to the distance from magnet isocenter in the *X*, *Y*, and *Z* directions, respectively. They therefore create a positionally dependent shift in the field and thus in the precessional frequency of spins in the sample. These linear overlays on the main field allow for all of the spin manipulations in frequency and phase performed in modern imaging and in vivo spectroscopy from frequency encoding to diffusion weighting.

### 3.2. Gradient Subsystem Components

A complete gradient insert consists of three orthogonal electromagnetic coils. Each of the axes, both the axial *Z* gradient and the transverse *X* and *Y* gradients, is wound with wire or etched from a copper-clad sheet and potted together as a single mechanical unit. The completed gradient is coaxially inserted into the resistive shim set or warm bore of the magnet. When energized, each coil creates a linear gradient in the *Z* component of the magnetic field with a minimal value at the isocenter and maximum positive and negative excursion at opposite ends of the designed field of view along the axis in question. Coils are defined by their maximum strength, set by the current they can carry, and their maximum rate of change or slew rate set by their inductance and the power of the driving gradient amplifier. Additional key parameters are field of view and linearity.

Several variations on the basic gradient implementation exist. Dedicated gradient sets for specific purposes are common on research systems. While typical clinical installations have a single, permanently inserted gradient insert that will admit a human torso, only head units are smaller and thus may have lower inductance and higher slew rates which is useful for specialized applications such as echo planar imaging. Unlike body gradients, units that admit only the head typically require an asymmetric design to permit a uniform linear gradient from the insertion end through the top of the subject's head. Another way to reduce the inductance seen by a driving amplifier and thus decrease the slew rate is to split the coil of a given axis into two complementary parts, each with their own gradient amplifier system. As the force on a pulsed electromagnet within a strong static magnetic field is substantial, torque compensated designs that counteract the moment with outer coils of opposite polarity are becoming standard.

The power electronics driving gradient coils are solid state constant current amplifiers designed specifically to supply the low resistance, very high inductance coils. They can supply high voltage as needed in order to overcome gradient coil inductance and allow rapid switching of gradient polarity. Gradient amplifiers must also supply high current in order to provide sufficient gradient strength for modern high speed imaging applications. Unlike the RF amplifiers, these amplifiers are designed to produce a low frequency direct current pulse at low duty cycles. As application of gradients precisely determines the phase acquired by spins, digital transmission of low voltage gradient waveforms from the spectrometer reduces the introduction of noise into the amplifier input. While early high performance gradient systems used a resonant system with amplifiers optimized for sine wave output at a particular frequency corresponding to the EPI readout, modern high performance gradients use more versatile amplifiers capable of arbitrary output waveforms.

## 4. RF Coils and Transceiver

Of all aspects of MRI engineering, radiofrequency coil, design, construction, and tuning are the most radically altered by higher frequencies. The coils used as transmit and receive elements in magnetic resonance research are fundamentally resonant LC circuits. As such, when excited at their resonant frequency, energy is alternately stored as electric field in the capacitors and magnetic field in the inductive elements of the circuit. The goal in NMR coil design is to minimize the generation of electric field near the subject while shaping the energy stored in the inductive portion of the circuit so that a homogeneous magnetic field is created in a direction transverse to the main magnetic field.

Within the gradient coils, there is an RF coil that produces the RF field and detects the received signal at and near the Larmor frequency. Since the frequency range used in MR imaging is the same as that used in radio and television broadcasting, the room housing the scanner is surrounded by an RF shield that prevents the RF pulses radiating outside the room and prevents other RF signals radiating into the scan room, Hornak [6]. The RF transmitter generates a high burst of RF energy (for a few milliseconds), and it then detects the received signal (which lasts from 10 to 1000 milliseconds).

**Figure 2:**
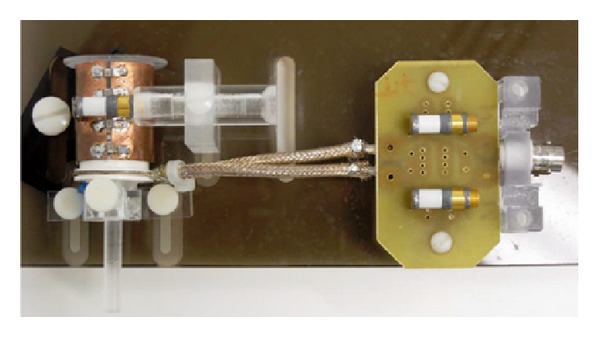
The RF coil and transceiver.

The same coil can be used for both transmitting and receiving a signal, in which case careful protection of the preamplifier in the receiving system is required from the instantaneous burst of energy during transmission. Use of separate coils for the transmitting and receiving processes has the advantage of individually optimizing the coil design for each function; however, care should be taken to prevent interaction since both coils operate at the same frequency.  Figure 2 illustrates the RF coil and transceiver. The fixed capacitors can be seen joining the two halves of the volume coil. There is also a variable balancing capacitor on top of the volume coil. The two other variable capacitors to the right match the loop's impedance to the loaded volume coil and input impedance of the transmitter/receiver.

The purpose of the transceiver is to produce the appropriate RF voltage to the RF coil and to detect the FIDs. During the receive state, the RF coils are connected to a low noise preamplifier and then to the main amplifier. Complex fast switching circuitry is required to protect the preamplifier from any signals from the transmission state from leaking and damaging it. The received signal is then digitized using analogue to digital (A/D) converters and then sent to the computer for processing.

RF coils for high field MRI operate, at 200 to 400 MHz, in the high VHF and low UHF range, thus straddling the technological boundary between antennas and klystron tubes. The design parameters of RF coils are unique, however, in that they are designed to create and/or receive a rapidly oscillating magnetic field within the coil volume rather than interact with an electromagnetic wave in the far field. Both homogeneous volume coils and inhomogeneous surface coils can be used for these purposes provided the appropriate RF pulses are used. Volume coils must efficiently transform the output of the power amplifier into a homogeneous magnetic field over the working volume of the coil without creating dangerous electric fields. Both surface and volume coils must be tuned broadly enough so that the frequency shift induced by different subject heads does not adversely affect the 50 ohm match. Yet they must also be tuned narrowly enough that the coil Q is high and the bandwidth of noise admitted is low.

Many designs of RF coil are possible, as detailed in the historical review below. The main categories have not changed, however. Volume coils are typically cylindrical and rely upon a sinusoidal distribution of currents arranged circumferentially around the tube and running the length of the coil to create a transverse magnetic field. Such coils as the birdcage and the TEM resonator generate a very homogeneous transverse fields within their field of view. Surface coils, in their simplest implementation, are formed from a partial loop of wire having dimensions such that its inductance resonates with the capacitance that completes the loop at the frequency of interest.

Simple surface coils and variations thereof have an inhomogeneous field profile that falls off away from the plane of the coil. Unlike volume coils, which are used primarily in transmit-receive and transmit only modes, the inhomogeneous field profile of surface coils restricts their use to the receive only mode except in the case where adiabatic pulses, Silver et al. [7], or trains of pulses, Robin Bendall and Gordon [8], are used to reshape the field profile. Despite their inhomogeneous field, surface coils are frequently used at high and low field because their close coupling to the sample leads to a higher signal to noise ratio than is a afforded by volume coils. Another method to gain signal to noise, used more frequently in volume coils but also possible with crossed surface coils, is quadrature reception. This method requires a fourfold axis of symmetry in the coil system. In quadrature coils, signals identical except for 90 degrees of phase difference are received by to orthogonal portions of the coil system. Since, once the extra phase shift is removed, the two signals are coherent but the noise received is incoherent, a net gain of sqrt(2) in SNR occurs, Chen et al. [9]. The use of circularly polarized coils also has the advantage of requiring half the power of a linearly polarized system in transmission. A further advantage of circular polarization and quadrature coils is improved homogeneity of the applied field, Glover et al. [10].

### 4.1. RF Coil Issues

High field coil design poses its own set of challenges quite distinct from RF coil issues at low field. The direct consequences of higher field strength are higher frequency and lower wavelength of the excitation energy. In addition to the effects of frequency on tissue penetration and inhomogeneity from dielectric resonances, the changes in frequency have great impact on design of the coils.

A primary high field RF coil design problem is avoiding coil self-resonance. The inductive elements in radiofrequency coils consist primarily of wires and strips of metal foil in simple linear and curved geometries. The inductance of these elements depends on their size and shape. Coil geometry in head coils is dictated by head size. Regardless of the particulars of the coil design, the basic size and shape dictate the inductance of the coil. Thus, the approximate inductance of a headcoil is the same at all field strengths and for all head shapes and sizes.

It can be seen that, for a fixed inductance, as the Larmor frequency increases, the capacitance must decrease. The lumped capacitances added to the coil are not the only source of capacitance in the coil, however. Due to the large number and surface area of parallel conductors in a typical volume coil design, there is a degree of capacitive coupling from one part of the coil to the next. The value of this capacitance, while small, can become significant at high field. Since capacitance adds in parallel, the total capacitance of a coil is not lower than the sum of the stray capacitance of the coil and the lumped capacitance added. If the appropriate lumped element capacitance for a particular frequency, as calculated above, becomes so small that its value is less than the stray capacitance of the coil, that stray capacitance will keep the resonant frequency lower than desired regardless of the value of the lumped capacitance. The only solution is to the change the coil geometry to lower the inductance. This has been part of the drive behind the evolution of coils outlined in the next section. The high stray capacitance is also the reason that high field coils are so difficult to tune and match: the sample itself will affect both the coil inductance and the coil capacitance. At low field, these effects are small as compared to the absolute value of coil inductance and capacitance while at high field; minor motion by the subject can change the tune and match significantly, Hyde et al. [11].

The concomitant decrease in wavelength at high field has effects on coil design as well. As discussed above, birdcage volume coils typically function by creating a sinusoidal distribution of current around the circumference of their end rings, Hayes et al. [12]. This distribution creates a transverse magnetic field. The birdcage coil is by far the most popular volume coil for brain imaging. In this coil, the sinusoidal current distribution is faithfully carried by the length of the coil by straight wires or metal foil. In the low frequency case the current distribution along these struts is relatively uniform. At high field, however, as the quarter wavelength of the exciting RF approaches the length of those longitudinal struts, they begin to act as a transmission line and take on a nonuniform current distribution along their length. The standing wave formed along the length of the coil results in a variation in the strength of the transverse magnetic field created in the head coil as a function of distance along the coil. Thus, flip angle in axial slices would no longer be constant; instead it would depend on the relative size of the coil compared to the quarter lambda length and the relative position of interest within the coil.

Changes in the Q of RF coils also affect their operation at high field. Q measures the efficiency with which the applied energy from the power amplifier is converted into magnetic field versus that portion which is lost as heat in the resistance of the coil or as radiation of RF from the coil. As the frequency increases, quality factor tends to decrease, perhaps in part because the skin effect increases resistance in loaded to unloaded quality factor. This quantity quantifies the proportion of energy that goes into the sample versus that lost to radiation and resistance. The coil structure itself, Robitaille [13], more important than absolute Q, however, is the ratio of Even as the efficiency of transfer increases however, the homogeneity of the B_1_ field is strongly influenced by the sample characteristics. As discussed in the section on dielectric resonances, these occur when the wavelength of the RF in a sample with a given dielectric constant approaches the dimensions of the sample.

The issues of coil homogeneity, self-resonance, coil efficiency, quadrature detection, transmission line effects, and quality factor have greatly affected RF coil design since the first solenoids. In turn, these designs have changed as Larmor frequencies have raised.

### 4.2. RF Receive and Transmit Subsystems

Routing of transmitted and received signals to and from the radio-frequency coil is accomplished by the RF front end. The goal of the RF front end on the transmit side is to transform the low voltage signal at the Larmor frequency with the appropriate envelope as generated in the spectrometer into a powerful pulse of RF energy which is efficiently delivered to the RF coil in quadrature without damaging the sensitive receive electronics. On the receiver side, the microvolt level signal from the RF coil must be immediately quad-detected and amplified before being sent to the spectrometer. The front end includes the initial amplification stages in the receive chain as well as the components which keep the transmitted and the received signals separate. Aside from the amplifier for the transmitted signal, all components of the front end are inside the magnet room, close to the RF coil. The front end consists of components on the transmit side, the receive side, and components that link the two sides.

The front end consists of a quadrature hybrid, a transmit-receive switch, and tuned low noise figure preamplifiers. Also included in this part of this system is the radio frequency power amplifier. The quadrature hybrid is a broadly tuned device that, when used with a quadrature coil, handles quadrature transmission and reception and contributes to the isolation between transmit and receive chains. Of the four ports on a quad hybrid, two ports are connected to a quadrature RF coil, the transmit port is connected to the RF power amplifier, and the receive port connects to the receiver chain. The two ports on the coil side are 90 degrees out of phase with each other. The transmit and receive ports are isolated (>50 dB) from one another such that transmitter power in the range of kilowatts will not damage sensitive receiver components designed to accept milliwatts. In addition to contributing to the isolation of the receive and transmit sides of the system, the hybrid is responsible for splitting the signal transmitted to the coil into two quadrature components in order to create a circularly polarized magnetic field in the coil. In the receive direction, the quadrature hybrid recombines two ports separated by 90 degrees of phase shift into one coherent signal with greater signal to noise than either channel alone. As such, only coils with fourfold symmetry that support quadrature drive use the quadrature hybrid. Rated power, port isolation, insertion loss, and frequency range are key specifications for a quad hybrid.

Between the quadrature hybrid and the preamplifiers, in the case of quadrature coils, or between the RF coil and the preamplifier, in the case of linearly polarized coil, lies the transmit receive (TR) switch. The TR switch is a tuned device which acts as a high power RF switch. One common configuration is a 3 port TR switch which functions as a single pole double throw switch. In the case of a linear drive coil that does not use a quad hybrid, one port receives the high power input from the RF amplifier, one port connects to the preamplifier, and the common port is connected to the coil. In this case, the switch connects the coil and transmits by default but switches to connect the coil and receiver side when gated to receive. For quadrature coils, the transmit signal enters the quadrature hybrid instead of the TR switch and so one port of a three-port switch is left terminated. The common port connects to the quadrature hybrid and the remaining port connects to the preamplifier. In its default position, during transmit, the switch connects the receive port of the quad hybrid to a high power terminator to absorb any transmitted signal not completely isolated.

In the receive position, the receive port of the quad hybrid is connected through to the preamplifier. Key specifications are insertion loss, isolation, rated power, and frequency range. The next step along the receive chain is the first amplification stage: the preamplifier. The preamplifier is designed to accept signals on the order of microvolts and amplify them on order of 10 to 100 fold. The noise figure of this first amplification stage is crucial because it sets the receive chain signal to noise. In a well designed RF front end, however, the total noise figure is dominated by the coil and sample rather than any part of the receiver chain. To achieve such high performance, tuned gallium arsenide field effect transistor based amplifiers are typically used.

The final piece of the RF transmit system is the RF power amplifier. RF power amplifiers take as their input a TTL blanking signal and a low voltage modulated carrier wave that is the RF pulse. They output a signal faithful in frequency, phase, and envelope to the input signal but with greatly increased power. This high power output travels through a low loss cable, through the penetration panel, and into the magnet room.

### 4.3. RF Engineering Issues

The issues associated with high field RF front end and power amplifier components revolve around the high frequency used and the high magnetic field to which the components are subject to. Cable loss is one ubiquitous problem at high field. At frequencies on the order of hundreds of megahertz, standard RG-58 coaxial cable loses more than 2 dB over 50 feet and more than 16 dB over 200 feet. Even hardline has substantial losses at these frequencies. The result is that gain stages for high field systems must be higher just to account for losses. The magnetic field itself presents a problem for some of the RF front end components that must be situated inside the magnet room. The quad hybrid, the TR switch, and the preamplifiers must all be able to operate properly in a field potentially on the order of several thousand Gauss. Since quadrature hybrids are passive devices typically based on microstrip designs, they are relatively insensitive to the field as long as no part of their construction uses ferromagnetic materials. TR switches, while actively controlled and pin-diode based, are likewise relatively unaffected by the field in which they operate.

Stock telecommunications preamplifiers, however, will not meet specifications unless modified for operation in the field. The tuned elements in the preamplifiers typically include an inductive portion, generally ferrite core inductor coils. When the iron ferrite is saturated by the ambient magnetic field, these inductors lose their designed inductance and thus change the tuning of the amplifier. Thus preamplifiers for use in NMR applications must use air core inductors.

The combination of high cable losses and a classically quadratic increase in required power for spin excitation adds up to very high output power requirements for the RF power amplifier. Hetherington et al. [14]. These amplifiers must produce tens of kilowatts at hundreds of megahertz without output droop or instability. Outside of MRI, power amplifiers as these frequencies use vacuum tube technology because solid state amplifiers typically cannot deliver more than a kilowatt of power. Tube amplifiers, however, are inefficient, relatively unstable, and unreliable at these frequencies, Vaughan et al. [15].

In addition to magnetic shielding, MRI systems require shielding from ambient electromagnetic waves. High field MRI systems operate at frequencies in the VHF and UHF broadcast range. As such, transmitted power from radio and television broadcasts will be received by the tuned coils and brought into the MRI receive system. These narrow band signals appear as zipper artifacts running the length of an image along the phase encode direction at the frequency of operation. To prevent this artifact and to prevent interference with surrounding radio and television equipment from operation of the MRI scanner, an RF shield is required. This shield is typically made up of copper sheeting or screening covering those areas not sealed by the iron used for magnetic shielding. The RF shield must be breached by at least one door for entry. Multiple technologies exist for creating a tight seal to RF when the door is closed: the simplest of which is conducting spring fingers all along its edge. More complicated arrangements include active sealing systems. The shield must also be breached by multiple cables for control lines, received signals, transmitted signals, room power, gradient lines, and so forth. These cables all pass through filtered connectors mounted on one of the shielded room's penetration panel.

## 5. Data Processing System

The data system can be divided into two parts. The first is the computer, which is responsible for many tasks such as acquiring data, generating pulse sequences, hardware control, patient table control, safety monitoring, image display, and so forth. The second part of the data system comprises the subsystems that, under the direction of the computer, perform specific functions.

### 5.1. Patient Handling and Monitoring

The patient handling system allows easy ingress and egress from the magnet and permits precise control in the axial direction of the positioning of the subject in the magnet. The patient monitoring system is a set of sensors used to monitor the patient for the purpose of safety and, for some applications, synchronization of imaging with physiological parameters.

Patient handling systems generally consist of a detachable patient table and a means for the sled resting on that table to be inserted into the magnet bore in a controlled fashion while the sled is docked to the magnet. Associated systems include bore lighting and air flow. Patient monitoring systems vary by manufacturer and the needs of a particular installation. Furthermore, whether a particular sensor is used to monitor a given patient depends on the type of investigation being performed. Typical monitoring systems include sensors for heart rate and rhythm, respiratory rate and rhythm, blood pressure, and blood oxygen saturation. Cardiac monitoring combines the use of a pulse oximeter with an electrocardiogram. Respiratory monitoring can be accomplished with either a bellows encircling the chest or an exhaled carbon dioxide meter fed via nasal cannula. Blood pressure monitoring may be done in the usual manner with an automatic oscillatory sphygmomanometer. Monitoring extends beyond awareness of changes in the subject's vital signs, however. Communication with the subject is an essential part of successful scanning. Audio intercom systems are necessary and video surveillance over and above direct monitoring through windows can be helpful.

The final component of subject monitoring is a component of the radiofrequency pulse transmission system: the power meter. The RF power meter measures the power output to the transmit coil. By monitoring and, if necessary, limiting the instantaneous output power over time according to medical guidelines, the power meter decreases the chance that joule heating of patient tissue will be significant. In addition, the magnet room has an oxygen sensor. Although unlikely, since gaseous helium is less dense than room air, in the event of a helium leak, the gas could displace the oxygen in the magnet room and therefore must be monitored for patient safety.

### 5.2. Issues

There are additional patient handling and safety monitoring concerns generated by high field systems over and above those for standard clinical systems. Unlike clinical systems under 4 T which the authorities have deemed minimal risk, continuous physiological monitoring of all subjects exposed to the 8 T field must be performed. While direct patient safety concerns are outlined in a separate chapter, risks to the patient due to equipment malfunction in the field must be addressed as well. In addition to the ballistic risk of attraction of any ferromagnetic equipment into the bore, the intense fringe fields present in the magnet room cause some devices, which at low field can be mounted just outside the bore, not to function properly. For example, the motor and drive electronics for the patient table must be well shielded and anchored far from the bore. In addition, the sensors and the control electronics for the patient monitoring equipment must be able to operate in the field. For devices whose sensor transmission lines are compatible with magnetic fields, such as fiber optic cable in the pulse oximeter or air lines in the blood pressure cuff, respiratory bellows, or carbon dioxide sensor, those lines can merely be extended in length in order to place the control electronics as far from the magnet as possible. Electrocardiogram units typically use instrumentation amplifiers in their control electronics connected via wires to electrodes placed on the patient. As such, the strong static magnetic field and intense oscillatory gradient and RF fields can induce significant noise in the connecting wires and may induce a voltage across the electrodes.

An issue specific to the patient table at high field is isolation from vibration. The magnetic field pulsations of the gradient system within the static field lead to Lorentz forces on the wires. The net result is that the entire gradient insert vibrates with each gradient pulse. Since the magnitude of this vibration is substantially greater at high field, isolation of the patient and RF coil from it is of greater necessity. Motion of the subject within the gradient coil due to the vibration will result in blurring of the image. One solution which allows significant isolation from the vibration is to cantilever the patient table. That is, the subject should be inserted into the bore on a sled that is nowhere in contact with the gradient or magnet bore. This requires a strong base on which to mount an extremely rigid sled. Due to the technical difficulties of this approach, they are only now becoming available.

### 5.3. Console Subsystem

Consoles are made up of a number of interlocking components that control every aspect of the magnetic resonance imaging experiment, Hoult [16]. Within the console lie the components which generate RF and gradient waveforms to be sent to their respective subsystems as well as the receiver and the control systems which pull them all together. In addition, the console includes the hardware which digitizes the received signal and decodes its spatial information to generate images.

Several components are essential to more than one part of the console. One such building block is the master frequency synthesizer. This is a high accuracy, low drift precision frequency generator with computer controlled frequency and stable phase. It is used to generate a signal at the Larmor frequency for generation of RF pulses and for downconversion of received signals. The arbitrary waveform generators are another general building block. They are digitally controlled flexible function generators which are used to supply the waveforms for the gradient subsystem as well as the envelope for RF pulses. As such, the gradient waveforms can be directly sent from the arbitrary waveforms generators to the gradient amplifiers. In the case of the RF amplifiers, the waveform generated by the arbitrary waveform generators are used to amplitude modulate the output of the master frequency synthesizer, either via direct mixing or a single upconversion step through an intermediate frequency, such that a high frequency pulse with a given envelope shape is sent to the RF amplifiers.

These quadrature mixers form another building block that is used both in generation of RF pulses with arbitrary phase as well as quadrature detection of the received signal. Control of the signal destined for the RF and gradient amplifiers rests with the pulse sequencing computer. This system coordinates the signal transmission, gradient pulsing, and receiver unblanking to microsecond tolerances. The receiver system takes the signal generated in the coil and amplified in the RF front end and processes it until it can eventually be converted into an image. The first step in this process is demodulation to remove the carrier, the Larmor frequency, such that only the audio frequency excursions remain.

Subsequently, phase sensitive detection is performed. Throughout these analog transformations, all mixing steps are followed by bandpass filtering to remove unwanted components and interspersed gain and attenuation stages ensure appropriate matching of signal to input dynamic range. Finally, the I and Q channels are digitized in the ADC and Fourier transformed. In general, both the I and Q data as well as the final image data are stored in computer memory. As analog to digital conversion chips gain in speed and performance, software radio architectures, in which the signal is digitized earlier in the process of reception, are on the horizon for MRI receiver design.

Traditional superheterodyne designs, however, are currently the standard. Once the data is acquired and available in the memory of the image processing and display computer, it can be stored on disk in an appropriate file format. Processing and storage of the raw data can be the rate limiting step in imaging as even fast Fourier transform algorithms are not instantaneous. In the case of functional MRI applications which may acquire 20 slices across the head every few seconds on a continuous basis for several minutes, either the amount of computer memory or the rate of archiving to the hard disk may limit the number of images that may be acquired. As in the earliest scanners working with multislice techniques, memory and permanent data storage is limited, Black et al. [4]. Nowadays, the memory and disc space is usually not a problem. There might still be a problem with the speed of data transfer. This is especially true of the huge matrices used in high resolution imaging at high field. A 2048 by 2048 image has 64 times the data of 256 by 256 image and therefore takes much longer to Fourier transform and requires much more disk space and great transfer speed.

### 5.4. Console Design Issues

Spectrometer designs, no matter the frequency of interest, require very tight tolerances in order that no phase errors occur at any point in the process of transmission or reception. The primary difference for high field systems is the downconversion stage. Demodulating the audio frequency data from the Larmor carrier may be done with the aid of more than one intermediate frequency due to the large gap in frequency. Once in the audio range, the receiver chain components of a high field spectrometer are no different than any other spectrometer. On the transmit side, the reverse is true. The modulator that shapes the RF pulses must be able to operate with a high frequency carrier input.

Research spectrometers in general and high field spectrometers in particular often have a number of other useful features. The first is multinuclear capability. This requires that the main frequency synthesizer be adjustable often over hundreds of megahertz and yet have accuracy to less than a cycle per second once set. The remaining modulators must likewise be able to handle the frequency range. Multiple receiver channels can be necessary for spectroscopy or imaging. The use of multichannel systems for multinuclear decoupling, polarization transfer, or proton localizers with low band imaging takes maximum advantage of console capability. In anatomical imaging, multiple channels are used for phased array imaging. They may also be necessary for applications such as SENSE which use multiple receiver coils.

## 6. Artifacts

**Figure 3:**
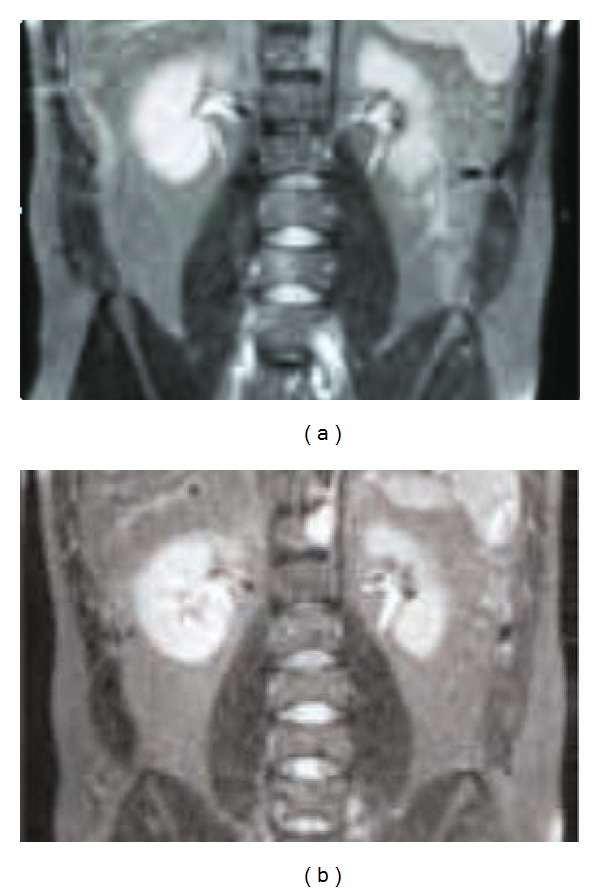
3 T system showing up to 40% improvement in SNR with direct digital (a) and signal loss resulting from digitization performed away from the coil (b).

Figure 3 depicts 3 T system showing up to 40% improvement in SNR with direct digital (a) and signal loss resulting from digitization performed away from the coil (b). MR imaging has developed rapidly in the last decade and has proven it to be a very reliable diagnostic tool. It is comparatively new to other existing imaging tools such as ultrasound and X-ray imaging and has therefore introduced new artifacts that need to be investigated. Artifacts can be defined as any aspect of an image which misrepresents the anatomic and geometrical relationships within the body, Atlas [17]. There are two important purposes for investigating artifacts and their sources: (1) to avoid false diagnoses being made based on artifacts, and (2) to learn how to eliminate them. Some of the artifacts and their sources can be easily identified, while some are difficult to interpret. The main sources artifacts are frequency shifts, sampling, truncation, and aliasing, instrumental errors, motion, noise, and measurement errors.

## 7. Conclusion

The major hardware manufacturers have learned what is required to turn temperamental precision lab equipment into robust high throughput machines for clinical field strengths. Higher fields, however, require considerable engineering from research groups to be successful. In the end, the quality of the implementation of an MRI system can be just as important as the particular field strength. That is, a poorly designed high field system may have lower signal to noise and more artifacts than a well optimized low field system. It is not surprising, therefore, that previous high field systems have undergone years of testing by experienced manufacturers before release to the research community. These research groups have had to go beyond the manufacturer's solution to fully optimize their high field systems. Before issues of RF penetration, dielectric resonances, and power can be addressed in full at a particular field strength, the system must be designed and built.
